# Gamma Visual Stimulation Induces a Neuroimmune Signaling Profile Distinct from Acute Neuroinflammation

**DOI:** 10.1523/JNEUROSCI.1511-19.2019

**Published:** 2020-02-05

**Authors:** Kristie M. Garza, Lu Zhang, Ben Borron, Levi B. Wood, Annabelle C. Singer

**Affiliations:** ^1^Neuroscience Graduate Program, Emory University, Atlanta, Georgia 30033,; ^2^Wallace H. Coulter Department of Biomedical Engineering, Georgia Institute of Technology and Emory University, Atlanta, Georgia 30332,; ^3^Parker H. Petit Institute for Bioengineering and Bioscience, Georgia Institute of Technology, Atlanta, Georgia 30332, and; ^4^George W. Woodruff School of Mechanical Engineering, Georgia Institute of Technology, Atlanta, Georgia 30332

**Keywords:** cytokines, gamma oscillations, neuroimmune signaling, neuroinflammation, NF-κB, phosphoproteins

## Abstract

Many neurodegenerative and neurological diseases are rooted in dysfunction of the neuroimmune system; therefore, manipulating this system has strong therapeutic potential. Prior work has shown that exposing mice to flickering lights at 40 Hz drives gamma frequency (∼40 Hz) neural activity and recruits microglia, the primary immune cells of the brain, revealing a novel method to manipulate the neuroimmune system. However, the biochemical signaling mechanisms between 40 Hz neural activity and immune recruitment remain unknown. Here, we exposed wild-type male mice to 5–60 min of 40 Hz or control flicker and assessed cytokine and phosphoprotein networks known to play a role in immune function. We found that 40 Hz flicker leads to increases in the expression of cytokines which promote microglial phagocytic states, such as IL-6 and IL-4, and increased expression of microglial chemokines, such as macrophage-colony-stimulating factor and monokine induced by interferon-γ. Interestingly, cytokine effects differed as a function of stimulation frequency, revealing a range of neuroimmune effects of stimulation. To identify possible mechanisms underlying cytokine expression, we quantified the effect of the flicker on intracellular signaling pathways known to regulate cytokine levels. We found that a 40 Hz flicker upregulates phospho-signaling within the nuclear factor κ-light-chain-enhancer of activated B cells (NF-κB) and mitogen-activated protein kinase (MAPK) pathways. While cytokine expression increased after 1 h of 40 Hz flicker stimulation, protein phosphorylation in the NF-κB pathway was upregulated within minutes. Importantly, the cytokine expression profile induced by 40 Hz flicker was different from cytokine changes in response to acute neuroinflammation induced by lipopolysaccharides. These results are the first, to our knowledge, to show how visual stimulation rapidly induces critical neuroimmune signaling in healthy animals.

**SIGNIFICANCE STATEMENT** Prior work has shown that exposing mice to lights flickering at 40 Hz induces neural spiking activity at 40 Hz (within the gamma frequency) and recruits microglia, the primary immune cells of the brain. However, the immediate effect of 40 Hz flicker on neuroimmune biochemical signaling was unknown. We found that 40 Hz flicker leads to significant increases in the expression of cytokines, key immune signals known to recruit microglia. Furthermore, we found that 40 Hz flicker rapidly changes the phosphorylation of proteins in the NF-κB and MAPK pathways, both known to regulate cytokine expression. Our findings are the first to delineate a specific rapid immune signaling response following 40 Hz visual stimulation, highlighting both the unique nature and therapeutic potential of this treatment.

## Abstract (precis)

Many neurodegenerative and neurological diseases are rooted in dysfunction of the neuroimmune system; therefore, manipulating this system has strong therapeutic potential. Prior work has shown that exposing mice to flickering lights at 40 Hz drives gamma frequency (∼40 Hz) neural activity and recruits microglia, the primary immune cells of the brain, revealing a novel method to manipulate the neuroimmune system.

## Introduction

Interactions between the brain and immune system play critical roles in neurological and neuropsychiatric disorders (Calsolaro and Edison, 2016; Wohleb et al., 2016). The neuroimmune system, including glial cells and immune signaling molecules, offers a unique target to treat disease and improve brain health. However, little is known about how to nonpharmacologically manipulate the immune system of the brain. Recent research has shown that exogenously stimulating neural electrical activity at gamma frequency (30–50 Hz), through sensory stimulation, promotes microglial activity, a component of the neuroimmune system, and decreases amyloid-β (Aβ), one of the key proteins that accumulate in Alzheimer's disease (AD; Iaccarino et al., 2016; Adaikkan et al., 2019). While this recent research shows that neural electrical activity manipulates a component of the neuroimmune system and has therapeutic implications, a broader understanding of how exogenous stimulation of neural activity affects neuroimmune function is required. Indeed, the immediate effects of exogenous gamma stimulation on biochemical signals that control immune function in the brain are unknown. While the previous literature in this area has focused on Alzheimer's disease pathology, it is important to understand these basic biological interactions between neural activity and immune function in healthy animals outside the context of AD. Furthermore, while 40 Hz visual stimulation is hypothesized to recruit the immune system, the effects of different frequencies of visual stimulation on immune signaling remains unknown. Determining how different frequencies of visual stimulation affect the immune system in the brain will enable new strategies to manipulate brain function.

Cytokines, extracellular soluble signaling proteins of the immune system, are a main communication signal between neurons and immune cells (Hanisch, 2002; Prieto and Cotman, 2017). Chemotactic cytokines, or chemokines, are responsible for attracting immune cells to a site of injury and proinflammatory and anti-inflammatory cytokines promote or reduce inflammation, respectively. While molecular signaling between neurons and microglia has been largely attributed to neuronal expression of the cytokine fractalkine (CX3CL1), which attenuates microglial overactivation (Sheridan and Murphy, 2013), many other cytokines may play a role in signaling between neurons and microglia. For example, glial-derived TNF-α is necessary for proper neuronal synaptic scaling and is also a modulator of microglia neuronal phagocytosis (Stellwagen and Malenka, 2006; Goshen et al., 2007; Neniskyte et al., 2014). In addition to regulating microglial activation and recruitment, cytokines are also involved in diverse neuronal and synaptic functions. Still, little is known about how neuronal electrical activity affects cytokine expression.

Cytokine expression and its effects on immune function are regulated by intracellular signaling, such as the nuclear factor κ-light-chain-enhancer of activated B cells (NF-κB) and the mitogen-activated protein kinase (MAPK) immunomodulatory pathways. These pathways are activated through the subsequent phosphorylation of proteins in a cascade, or phospho-signaling, and they culminate in the activation of transcription factors, which regulate cytokine transcription, expression, and release from the cell. Both the NF-κB and MAPK immunomodulatory pathways strongly regulate the expression of diverse cytokines involved in immune responses, like microglial activation and recruitment [e.g., macrophage-colony-stimulating factor (M-CSF), MCP-1; Hommes et al., 2003; Cloutier et al., 2007; Liu et al., 2017]. These pathways also control the expression of neurotrophic and synaptotrophic factors and are involved in mechanisms of learning and memory (Sweatt, 2001; Kaltschmidt et al., 2006; Mattson and Meffert, 2006; Chen et al., 2017). Recent studies have shown 40 Hz flicker changes protein phosphorylation patterns after weeks of exposure compared with no stimulation, but the immediate effect of flicker on protein phosphorylation in general and the immediate effects of flicker on these two key phosphoprotein pathways are still unknown (Adaikkan et al., 2019).

In the present study, we determined how 40 Hz visual stimulation affects expression of key cytokines involved in the immune response, synaptic plasticity, and neuronal health of the brain. Furthermore, since cytokine expression is regulated by intracellular signaling, we assessed whether NF-κB and MAPK phospho-signaling changes before cytokine expression in mice exposed to 40 Hz visual stimulation compared with control visual stimuli. We tested this by exposing mice to varying durations of light-emitting diode (LED) light strips flickering at 40 Hz, which is known to induce gamma neuronal activity, as well as several control conditions (Gray et al., 1989; Iaccarino et al., 2016; Singer et al., 2018; Adaikkan et al., 2019). Our analysis showed that 1 h of 40 Hz flicker stimulation induces phospho-signaling within the NF-κB and MAPK pathways (∼15–60 min) and protein expression of diverse cytokines. Interestingly, 20 Hz flicker, random flicker, and constant light each induced unique cytokine expression profiles, revealing that different frequencies of visual stimulation induce unique immune signaling patterns. We then determined whether the cytokine response after 40 Hz flicker differed from that due to a model of acute inflammation induced by lipopolysaccharide (LPS) administration. We found that the cytokine profile of acute inflammation due to LPS administration was distinct from the cytokine response to 40 Hz flicker. These results show that 40 Hz flicker induces NF-κB phospho-signaling followed by MAPK phospho-signaling and increased cytokine expression distinct from inflammation. Importantly, the cytokines assessed here, as well as the NF-κB and MAPK pathways, play key roles in many different cellular functions. Furthermore, different frequencies of stimulation induced unique cytokine expression patterns. Thus, our results suggest that the stimulation of different patterns of activity may be used to regulate the expression of genes that promote neuronal health, synaptic plasticity, and healthy immune activity.

## Materials and Methods

### 

#### 

##### Animals.

All animal work was approved by The Georgia Tech Institutional Animal Care and Use Committee. Adult (2- to 3-month-old) male C57BL/6J mice were purchased from The Jackson Laboratory. Mice were pair housed upon arrival and allowed to acclimate to the environment for at least 5 d before being handled.

For all animals, food and water were provided *ad libitum*. For 3 d before the experiment, mice were single housed and briefly handled (∼1 min/mouse). All experiments were performed during the light cycle and began between 8:00 and 9:00 A.M. For experiments with flicker durations of <1 h, animals undergoing the same stimulation condition were interspersed with animals undergoing different conditions, to ensure circadian rhythms did not impact results. For experiments with a duration of flicker exposure at 1 h, we conducted multiple experiments, at different times of day, with a varying order of stimulus presentation, to ensure results remained consistent (data not shown).

##### Visual stimulation exposure.

To habituate and reduce visual stimulation, mice were placed in a dark room in the laboratory, for at least 1 h, before beginning each experiment. To commence the experiment, mice were transferred from their home cage to a similar cage without bedding, termed a flicker cage. The flicker cage was covered in dark material on all but one side, which was clear and faced a strip of LED lights. Animals remained in the dark room, where they were exposed to either LED lights flashing at 40 Hz frequency (12.5 ms light on, 12.5 ms light off), 20 Hz frequency (25 ms light on, 25 ms light off), a random frequency that averaged at 40 Hz (12.5 ms light on, variable duration light off), or constant light on stimulation, using a dimmer to ensure the same total luminance (∼150 lux) as a 40 Hz light flicker (Movie 1; Singer et al., 2018). These conditions allowed us to control for light stimulation (light), light stimulation flashes (random), and frequency-specific stimulation (20 Hz). Animals were exposed to visual stimulation for either 5 min, 15 min, or 1 h.

Movie 1. Example video of mouse undergoing flicker stimulation. Note, this video shows 20 Hz flicker because 40 Hz flicker is blurred with standard movie frame rates. 10.1523/JNEUROSCI.1511-19.2019.video.1

Immediately after stimulation exposure, mice were anesthetized with isoflurane, and within 3 min mice were decapitated and brains were removed. The visual cortex of the left hemisphere was microdissected, placed in an Eppendorf tube, and flash frozen using liquid nitrogen.

Cytokine analysis was conducted using six animals per group. Phosphoprotein experiments were conducted and combined across several cohorts of animals, using a total of 72 animals. More animals were used for experiments examining 5 min of stimulation due to concern that initial signals at this time point may be transient and hard to detect.

##### Lipopolysaccharide stimulation.

Animals were intraperitoneally injected with 5 mg/kg LPS (*n* = 6), diluted in saline (lot #039M-4004V, Sigma-Aldrich) or saline (vehicle; *n* = 5). Three hours after injection, mice were anesthetized with isoflurane, and, within 3 min, mice were decapitated and brains were removed. The visual cortex of the left hemisphere was microdissected, placed in an Eppendorf tube, and flash frozen using liquid nitrogen. Cytokine analysis was conducted as described below.

##### Cytokine and phosphoprotein assays.

For signaling and cytokine analysis, the visual cortex was thawed on ice and lysed using Bio-Plex Lysis Buffer (Bio-Rad). After lysing, samples were centrifuged at 4°C for 10 min at 13,000 rpm. Protein concentrations in each sample were determined using a Pierce BCA Protein Assay (Thermo Fisher Scientific). Total protein concentrations were normalized in each sample using Bio-Plex Lysis Buffer (Bio-Rad). For MAPK and NF-κB pathway analysis, 1.5 μg of total protein was loaded, and 6 μg was loaded for cytokine analysis. Preliminary experiments determined these protein concentrations fell within the linear range of bead fluorescent intensity versus protein concentration for detectable analysis. Multiplexed phosphoprotein analysis was conducted by adapting the protocols provided for the Milliplex MAP MAPK/SAPK Signaling Magnetic Bead 10-Plex [phospho (p)-ATF2, p-Erk, p-HSP27*, p-JNK, p-c-Jun, p-MEK1, p-MSK1, p-p38*, p-p53*] Kit and the MILLIPLEX MAP NF-κB Signaling Magnetic Bead 6-Plex Kit (c-Myc*, p-FADD, p-IκBα*, p-IKKα/β, p-NFκB, TNFR1). Cytokine analysis was conducted by adapting protocols provided for the MILLIPLEX Mouse MAP Mouse Cytokine/Chemokine Magnetic Bead Panel 32-Plex Kit [eotaxin, granulocyte (G)-CSF, granulocyte macrophage (GM)-CSF, interferon-γ (IFN-γ), IL-1α, IL-1β, IL-2, IL-3, IL-4, IL-5, IL-6, IL-7, IL-9, IL-10, IL-12p40, IL-12p70, IL-13, IL-15, IL-17, IP-10, KC (CXCL1), LIF, LIX, MCP-1, M-CSF, monokine induced by interferon-γ (MIG), MIP-1α, MIP-1β, MIP-2, RANTES (regulated on activation, normal T cell expressed and secreted), TNF-α, and VEGF]. All kits were read on a MAGPIX System (Luminex). Asterisks denote analytes that did not fall within the linear range and were therefore excluded from our analysis.

##### Partial least-squares discriminant analysis.

Data were *z*-scored before analysis. A partial least-squares discriminant analysis (PLSDA) was performed in MATLAB (MathWorks) using the algorithm obtained from the Mathworks File Exchange by Cleiton Nunes (MathWorks; Eriksson et al., 2006). To identify latent variables (LVs) that best separated conditions, an orthogonal rotation in the plane of the first two latent variables (LV1–LV2 plane) was performed. Error bars for LV1 figures represent the mean and SDs after iteratively excluding single samples (a leave-one-out cross-validation), one at a time, and recalculating the PLSDA 1000 times.

To test different flicker conditions and durations, phosphoprotein experiments were performed over several cohorts, and samples were normalized by removing batch effects. We performed a batch-effects analysis (limma package in R version 3.5.2) to remove any between-experiment variability before conducting the PLSDA. Outliers were removed by performing a principle component analysis on the data and iteratively removed data points that fell outside of a 99.5% confidence ellipse (mahalanobisQC in R version 3.5.2).

##### Animal behavior assays.

To analyze animal behavior, mice were placed in a polycarbonate cage (210 × 375 × 480 mm), covered on all sides but one with 100% polyester black material (similar to cage in the visual flicker experimental protocol cage described above, but larger and without a cage top). To record the mice in the dark, an infrared (IR) light was placed above the cage. To record mice, without the flicker stimulation affecting behavior tracking, a Basler Ace monochrome IR-sensitive camera with a Gigabit Ethernet interface with attached IR-only filter was used for recording. Ethernet connection from the camera to the computer allowed for Ethovision to record and analyze the experiment in real time.

Three days before recording, mice were handled as described in the flicker experiment section above. On the third handling day, each mouse was placed in the behavior cage to habituate for 5 min. On the fourth day, each mouse was recorded individually for 1 h and 5 min while receiving one of four flicker treatments (40 Hz, 20 Hz, random, light). Behavior assays were conducted using a total of six animals, with a within-subjects design. Each mouse received each treatment separated by 1 d in a randomized order. Experimenter was blinded to treatment type during experiment and analysis.

Ethovision XT version 14.0 was used to track and analyze behavior. First, the arena was defined and a zone group was defined that split the cage into two halves, front and back. A second zone group defined as center consisted of half of the total area in the center of the cage. Detection settings were made using the automatic detection function of Ethovision and were adjusted after preliminary analysis. Automated video analysis recorded the center point of the animals, and all recordings were checked and manually corrected through software interpolation for any time points in which the mouse could not be automatically detected. Activity level was designated as active or inactive, with inactive indicating freezing. Inactivity was determined to be <0.01% of the total arena having activity and lasting for a duration longer than half a second. Data for each variable were exported and analyzed using GraphPad Prism (GraphPad Software). A one-way ANOVA was conducted to assess differences between groups for each variable of interest.

##### Experimental design and statistical analysis.

Animals were randomly assigned to flicker exposure groups, and experimenters were blind to flicker exposure conditions during analysis for all experiments. Sample sizes were determined on preliminary data with a 0.80 power and an α of 0.05, and samples sizes were adjusted based on high variability. Control groups (20 Hz, random, and light) were based on prior experiments and preliminary data.

For multiplex analysis, either a one-way ANOVA (more than two groups) or a two-tailed unpaired *t* test (two groups) was used to determine whether there was a significant LV1 separation between groups. The top correlated cytokines and/or phosphoproteins on the LV1 were isolated, and an ANOVA or two-tailed unpaired *t* test was performed using GraphPad Prism 8 (GraphPad Software) to determine statistical significance between groups. These tests were followed by a *post hoc* Dunnett's multiple-comparisons test to determine differences between specific groups or a Tukey's multiple-comparisons test to compare differences in phosphorylation levels across time. Levels of significance were set to *p < 0.05, **p < 0.01, ****p < 0.001.

To further confirm significant differences between groups using PLSDA, a permutation analysis was conducted, which randomly assigned animals into experimental groups and ran the PLSDA based on these shuffled values 1000 times (Golland et al., 2005). For each test, true group assignment showed *p*_permute_ < 0.05 compared with the randomly permuted distribution, further confirming the validity of our data.

For analyzing changes in animal behavior, a one-way repeated-measures ANOVA (RM-ANOVA) was used to compare results between different stimulation types.

For comparing between LPS-treated and 40 Hz-stimulated animals, each group was normalized to control. Specifically, each cytokine level in each animal was divided by the mean cytokine level in the related control group (vehicle for LPS and random for 40 Hz). Multiple *t* tests were then conducted to compare each stimulation to its control; multiple comparisons were corrected using the Holm–Sidak method.

##### Code accessibility.

Data were analyzed using a custom code that is available upon request.

## Results

### 40 Hz flicker induces increased cytokine profile

**Figure 1.**
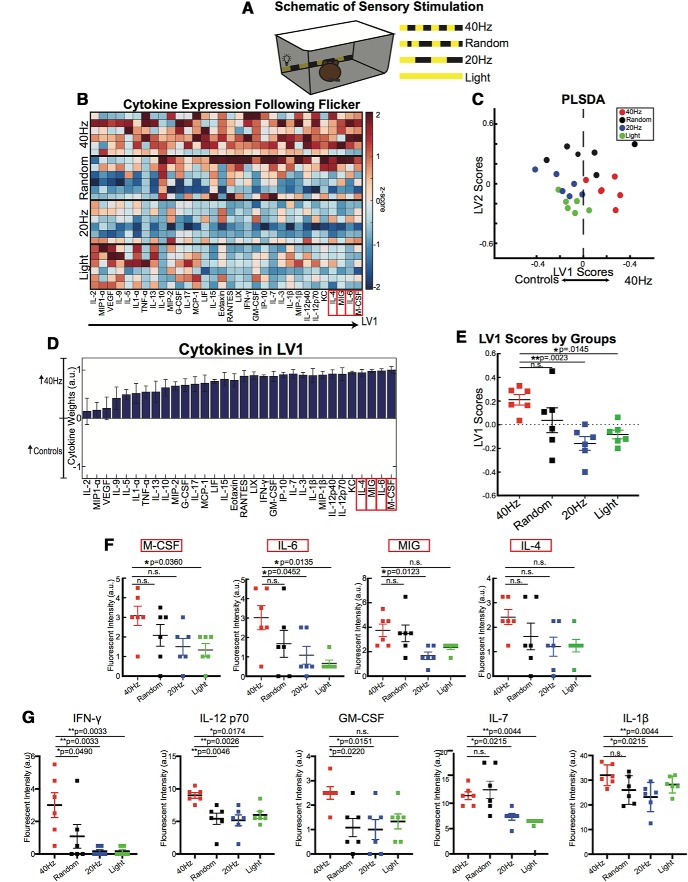
One hour of 40 Hz flicker increased cytokine expression in visual cortex. ***A***, Experimental configuration for presenting visual stimulation. ***B***, Cytokine expression in visual cortices of mice exposed to 1 h of visual stimulation. Each row represents one animal (*n* = 6). Cytokines (columns) are arranged in the order of their weights on the LV1 in ***D***. Color indicates *z*-scored expression levels for each cytokine. Top four cytokines from LV1 are boxed in red. ***C***, PLSDA identified LV1, the axis that separated 40 Hz flicker-exposed animals (red) to the right, 20 Hz flicker (blue) and constant light (green)-exposed animals to the left and random (black) flicker-exposed animals toward the middle (dots indicate individual animals for all graphs in this figure). LV2 separated 20 Hz, random, and light conditions. ***D***, The weighted profile of cytokines that make up LV1 based on which cytokines best correlated with separation of 40 Hz (positive) versus flicker control groups (negative). (mean ± SD from a leave-one-out cross-validation). ***E***, LV1 scores were significantly different for the 40 Hz group compared with controls (mean ± SEM; *F*_(3,20)_ = 5.855, *p* = 0.0049, one-way ANOVA). Each dot represents one animal. Results were confirmed with shuffling analysis (see Materials and Methods). ***F***, There were significant differences in expression of most of the top four analytes, M-CSF, IL-G, MIG, and IL-4 when assessed individually. The *p* values for comparisons between groups are listed in the figure. For one-way ANOVA comparisons across all groups: M-CSF: *F*_(3,20)_ = 2.970, *p* = 0.0564; IL-6: *F*_(3,20)_ = 3.775, *p* = 0.0269; MIG: *F*_(3,20)_ = 4.532, *p* = 0.0140; and IL-4: *F*_(3,20)_ = 2.051, *p* = 0.1391. ***G***, There were significant differences in the expression of IFN-γ, IL-12p70, GM-CSF, IL-7, and IL-1β as well. The *p* values for comparisons between groups are listed in the figure. For one-way ANOVA comparisons across all groups: IFN-γ: *F*_(3,20)_ = 6.32, *p* = 0.0034; IL-12p70: *F*_(3,20)_ = 6.428, *p* = 0.0032; GM-CSF: *F*_(3,20)_ = 4.13, *p* = 0.0197; IL-7: *F*_(3,20)_ = 9.541, *p* = 0.0004; and IL-1β: *F*_(3,20)_ = 3.404, *p* = 0.0377. SEM, standard error of the mean.

Given previous findings showing that exposing mice to 40 Hz light flicker leads to morphological changes in microglia, we speculated that 40 Hz flicker affects neuroimmune signaling. Specifically, since cytokines are key regulators of immune activity in the brain, we hypothesized that 40 Hz light flicker promotes cytokine expression in the visual cortex. To test this, we exposed animals to 1 h of 40 Hz flickering light, light flickering at a random interval averaging to 40 Hz (random), 20 Hz flickering light (20 Hz), and constant light (light; Fig. 1*A*, Movie 1). After visual stimulation, mice were sacrificed, and the visual cortex was rapidly (<3 min) microdissected and flash frozen. Using a Luminex multiplexed immunoassay, we quantified expression levels of 32 cytokine proteins in the visual cortex (Fig. 1*B*). To account for the multidimensional nature of the data, we used a PLSDA to identify profiles of cytokines that distinguished the effects of 40 Hz stimulation from the control groups (Fig. 1*C*; Eriksson et al., 2006).

**Figure 2.**
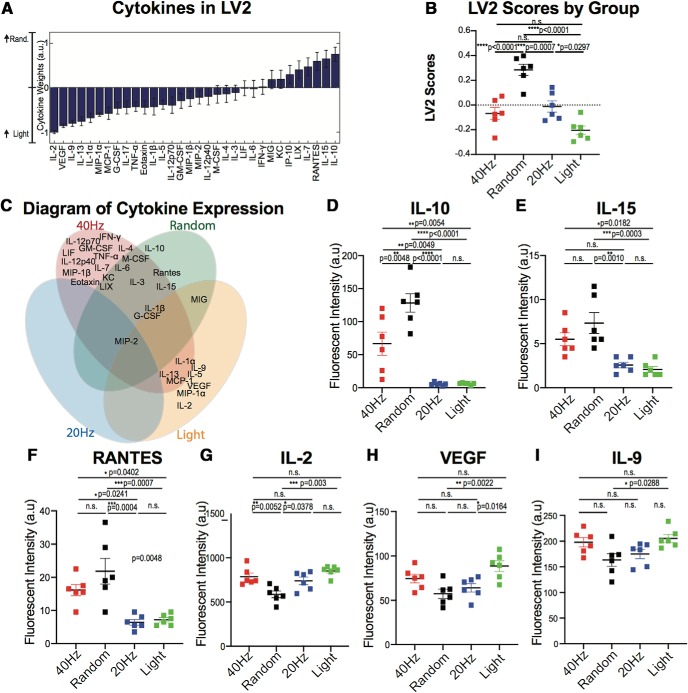
Flicker control conditions each lead to unique cytokine expression. ***A***, The weighted profile of cytokines that make up LV2 based on which cytokines best correlated with separation of random (positive) versus 20 Hz versus light flicker control groups (negative; mean ± SD from a leave-one-out cross-validation). ***B***, LV2 scores were significantly different for all four groups (mean ± SEM; *F*_(3,20)_ = 21.77, *p* < 0.0001, one-way ANOVA). Each dot represents one animal. The *p* values from Tukey's multiple-comparison test are listed. ***C***, Diagram of cytokine expression, with each cytokine name arranged based on increased expression per stimulation group. ***D***, One-way ANOVA displays significant difference in IL-10 expression across groups (mean ± SEM; *F*_(3,20)_ = 27.29, *p* < 0.0001, one-way ANOVA). The *p* values from Tukey's multiple-comparison test are listed. ***E***, As in ***D*** for IL-15 expression across groups (mean ± SEM; *F*_(3,20)_ = 11.33, *p* = 0.0001, one-way ANOVA). ***F***, As in ***D*** for RANTES expression across groups (mean ± SEM; *F*_(3,20)_ = 11.48, *p* = 0.0001, one-way ANOVA). ***G***, As in ***D*** for IL-2 expression across groups (mean ± SEM; *F*_(3,20)_ = 9.366, *p* = 0.0005, one-way ANOVA). ***H***, As in ***D*** for VEGF expression across groups (mean ± SEM; *F*_(3,20)_ = 6.790, *p* = 0.0024, one-way ANOVA). ***I***, As in ***D*** for IL-9 expression across groups (mean ± SEM; *F*_(3,20)_ = 4.076, *p* = 0.0207, one-way ANOVA). SD, standard deviation.

We observed overall higher cytokine expression levels across animals exposed to 40 Hz flicker compared with other groups. A PLSDA identified a LV1, consisting of a weighted linear combination of cytokines, as being most upregulated in 40 Hz (higher LV1 scores) or from all control conditions (negative; lower LV1 scores; Fig. 1*D*). LV1 best separated 40 Hz from all control groups (Fig. 1*C*,*D*). LV1 scores differed significantly between groups, confirming that the groups were statistically separate, specifically, 40 Hz flicker versus light and 20 Hz stimulation were best separated (*F*_(3,20)_ = 5.855, *p* = 0.0049, one-way ANOVA; Fig. 1*E*). LV2 significantly separated control groups from each other (*F*_(3,20)_ = 21.77, *p* < 0.0001; Figs. 1*C*, 2*B*).

The top four identified by the PLSDA to contribute to differences between groups were M-CSF, IL-6, MIG, and IL-4 (Fig. 1*F*). Of these top upregulated cytokines, IL-6 and MIG were significantly different between groups when each one was analyzed individually (IL-6: *F*_(3,20)_ = 3.775, *p* = 0.0269; MIG: *F*_(3,20)_ = 4.532, *p* = 0.0140, one-way ANOVA; Fig. 1*F*). We found that IL-6 had significantly higher expression in 40 Hz than in 20 Hz flicker and light; and MIG had significantly higher expression in 40 Hz than in 20 Hz flicker (IL-6: 40 vs 20 Hz: mean difference = 1.917, adjusted *p* = 0.0452; IL-6: 40 Hz vs light: mean difference = 2.33, adjusted *p* = 0.0135; MIG: 40 vs 20 Hz: mean difference = 2.083, adjusted *p* = 0.0123, Dunnett's test). M-CSF expression was higher in 40 Hz flicker than in light-exposed animals (mean difference = 1.750, adjusted *p* = 0.0360, Dunnett's test). We found no significant differences in IL-4 between groups, although expression levels trended toward being higher in 40 Hz flicker animals (*F*_(3,20)_ = 2.051, *p* = 0.1391, one-way ANOVA). Based on observed differences between groups in a heatmap of these cytokines (Fig. 1*B*), we ran additional analysis on a subset of cytokines and found significant differences in IL-12p70, IL-1β, IFN-γ, GM-CSF, and IL-7 between 40 Hz flicker and control conditions (Fig. 1*G*). These findings show that 1 h of 40 Hz flicker drives a significant neuroimmune signaling response, and this response is distinct from other flicker frequencies or exposure to constant light.

Interestingly, while animals exposed to 40 Hz flicker showed a higher cytokine expression profile, we also observed distinct cytokine expression profiles for random, 20 Hz, and light stimulation conditions (Figs. 1*B*, 2*C*). Indeed, our analysis revealed a second latent variable (LV2), which best separated these three control groups (Figs. 1*C*, 2*A*). Quantification of these effects showed that constant light leads to an upregulation of a subset of cytokines, unique from those upregulated by 40 Hz flicker. For example, when analyzed individually, IL-2, VEGF, IL-9, IL-1α, and IL-13 all showed significant differences in expression levels between groups, with higher upregulation after light exposure than at least one other group (IL-2: *F*_(3,20)_ = 9.366, *p* = 0.0005; VGEF: *F*_(3,20)_ = 6.790, *p* = 0.0024; IL-9: *F*_(3,20)_ = 4.076, *p* = 0.0207; IL-1α: *F*_(3,20)_ = 4.399, *p* = 0.0157; IL-13: *F*_(3,20)_ = 3.802, *p* = 0.0263; one-way ANOVA; Dunnett's multiple-comparisons method was used to control for three comparisons; Fig. 2*E*,*I*). In contrast, random stimulation uniquely upregulated IL-10 across all samples, when compared with other stimulation types (*F*_(3,20)_ = 27.29, *p* < 0.0001; Fig. 2*D*). These results highlight that while 40 Hz flicker modulated cytokine activity, 20 Hz, random, and light exposure also led to distinct cytokine activity.

### 40 Hz flicker induces NF-κB and MAPK signaling

**Figure 3.**
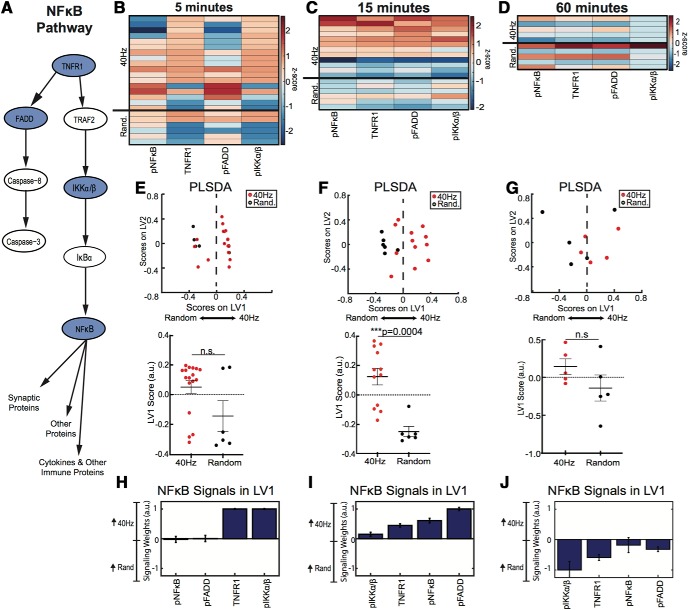
NF-κB phosphoprotein signaling in visual cortex increased in response to 15 min of 40 Hz flicker. ***A***, Simplified diagram of NF-κB signaling including phosphoproteins quantified in the present study (blue ovals). ***B***, NF-κB phosphoproteins quantified in visual cortex in mice exposed to 5 min of 40 Hz or random flicker (*z* scored). Each row represents one animal with groups separated by thicker black horizontal lines. Twenty-three total animals were analyzed, *n* = 17 in 40 Hz group, *n* = 6 in random group; outliers removed through a principle component analysis on the data and iterative removal of data points outside a 99.5% confidence ellipse (mahalanobisQC in R version 3.5.2). ***C***, As in ***B*** for mice exposed to 15 min of 40 Hz or random flicker; 40 Hz, *n* = 12; random, *n* = 6. ***D***, As in ***B*** for mice exposed to 60 min of 40 Hz or random flicker; 40 Hz, *n* = 6; random, *n* = 6. ***E***, Top, PLSDA of phospho-signaling data from mice exposed to 5 min of flicker, separating 40 Hz (red)-exposed animal to the right and random (black) to the left, along LV1. Bottom, Plots of LV1 scores between groups show a trend toward higher LV1 scores in 40 Hz-exposed animals (difference between means ± SEM = −0.1943 ± 0.09455; *t*_(21)_ = 2.065, *p* = 0.0525, two-tailed *t* test). Dots indicate individual animals for all graphs in this figure. ***F***, Top, As in ***E*** for mice exposed to 15 min of 40 Hz or random flicker. Bottom, Plot of LV1 scores between groups reveals significantly higher LV1 scores in 40 Hz-exposed animals (difference between mean ± SEM values = −0.3721 ± 0.08351; *t*_(16)_ = 4.456, *p* = 0.0004, two-tailed *t* test). Results were confirmed with permutation analysis (see Materials and Methods). ***G***, Top, As in ***E*** for mice exposed to 60 min of 40 Hz or random flicker. Bottom, Plot of LV1 scores between groups shows no significant difference (difference between mean ± SEM values = −0.2829 ± 0.2; *t*_(8)_ = 1.415, *p* = 0.1948, two-tailed *t* test). ***H***, The weighted profile of NF-κB phosphoproteins that make up LV1 based on which phosphoproteins best correlated with 40 Hz (positive) or random (negative) after 5 min of flicker exposure (mean ± SD from a leave-one-out cross-validation). pIKKα/β and TNFR1 most strongly contributed to separation between the groups. ***I***, As in ***H*** for 15 min of flicker. pFADD expression in 40 Hz flicker group and pNF-κB in random group most strongly contributed to separation between groups. ***J***, As in ***H*** for 60 min of flicker.

**Figure 4.**
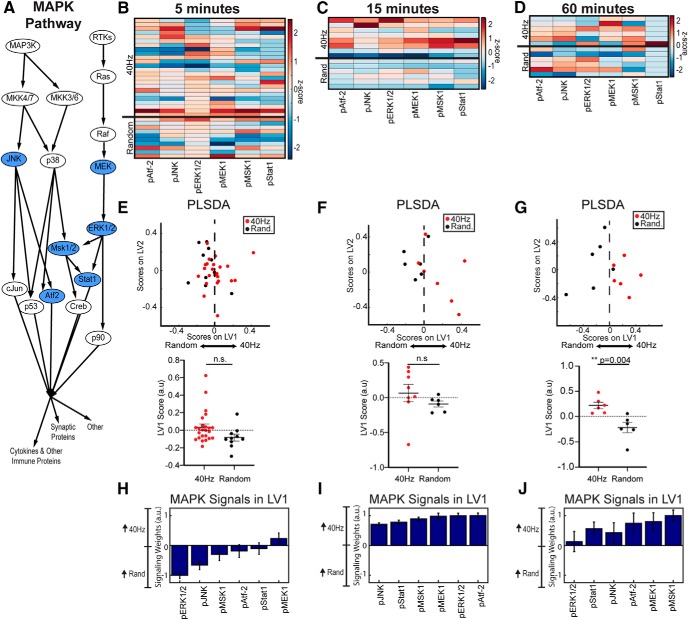
MAPK phosphoprotein signaling in visual cortex increased in response to 60 min of 40 Hz flicker. ***A***, Simplified diagram of MAPK signaling including phosphoproteins quantified in the present study (blue ovals). ***B***, MAPK phosphoproteins quantified in visual cortex in mice exposed to 5 min of 40 Hz or random flicker (*z* scored). Each row represents one animal with groups separated by thicker black horizontal lines. Thirty-four total animals were analyzed, *n* = 24 in 40 Hz group, *n* = 10 in random group; outliers were removed through a principle component analysis on the data and iterative removal of data points outside a 99.5% confidence ellipse (mahalanobisQC in R version 3.5.2). ***C***, As in ***B*** for mice exposed to 15 min of 40 Hz or random flicker; 40 Hz, *n* = 8; random, *n* = 6. ***D***, As in ***B*** for mice exposed to 60 min of 40 Hz or random flicker. ***E***, Top, PLSDA on phospho-signaling data for mice exposed to 5 min of flicker did not separate 40 Hz (red) samples and random (black) along LV1. Bottom, Plot of LV1 scores per group shows no significant differences between groups (difference between mean ± SEM values = −0.1181 ± 0.06318; *t*_(32)_ = 1.870, *p* = 0.0707, unpaired two-tailed *t* test). Dots indicate individual animals for all graphs in this figure. ***F***, Top, As in ***E***, for mice exposed to 15 min of 40 Hz or random flicker. Bottom, Plot of LV1 scores per group reveals a nonsignificant increase in 40 Hz LV1 expression in 40 Hz-exposed animals. (−0.1572 ± 0.1311; *t* (8.582) = 1.199, *p* = 0.2624, unpaired *t* test with Welch's correction). ***G***, Top, As in ***E*** for mice exposed to 15 min of 40 Hz or random flicker. Bottom, Plot of LV1 scores per group shows significantly higher LV1 scores in 40 Hz-exposed animals (−0.4393 ± 0.1185; *t*_(10)_ = 3.709, *p* = 0.0040, two-tailed *t* test). ***H***, The weighted profile of MAPK phosphoproteins that make up LV1 based on which best correlated with 40 Hz (positive) or random (negative) flicker after 5 min of flicker exposure (mean ± SD from a leave-one-out cross-validation). ***I***, As in ***H*** for 15 min of flicker. ***J***, As in ***H*** for 60 min of flicker. pMSK and pMEK expression in the 40 Hz flicker group most strongly contributed to separation between groups.

Cytokine expression is regulated by several canonical intracellular phospho-signaling pathways, including the NF-κB and MAPK pathways. Thus, to gain better insight into intracellular signaling changes that underlie changes in cytokine expression after visual stimulation, we examined how 40 Hz flicker impacted these two phosphoprotein pathways. Because phospho-signaling is usually transient, we examined this signaling after 5, 15, and 60 min of 40 Hz and random flicker (Janes et al., 2005). We focused on random flicker as our control group for this comparison specifically because it controls for the average number of times the lights turn on and off, the percentage of time the light is on, and the frequency of light presentation without inducing periodic neural activity (Iaccarino et al., 2016; Singer et al., 2018; Adaikkan et al., 2019). As with cytokine experiments, immediately following flicker exposure, mice were sacrificed, and visual cortex was rapidly microdissected. We used Luminex assays and PLSDA to quantify differences in the NF-κB and MAPK phosphoprotein pathways in animals exposed to 40 Hz versus random flicker exposure after different durations of flicker (Figs. 3, 4).

We found that phospho-signaling in the NF-κB pathway was significantly upregulated after 15 min, but not 5 or 60 min, of 40 Hz versus random flicker. Experiments were performed across several cohorts and animals were removed based on an outlier analysis; data were *z* scored according to batch-corrected data (see Materials and Methods). Our analysis revealed a trend of increased phosphorylation after 5 min of flicker, driven specifically by increases in TNFR1 and pIKKα/β, but 40 Hz and random groups were not significantly different (*t*_(21)_ = 2.056, *p* = 0.0525, unpaired *t* test; Fig. 3*B*,*E*). After 15 min of flicker exposure, NF-κB phosphorylation significantly differed between groups, as indicated by significant separation of NF-κB LV1 scores (*t*_(16)_ = 4.456, *p* = 0.0004,unpaired *t* test; Fig. 3*C*,*F*). At 60 min, there was no significant difference between groups, but phosphorylation of NF-κB proteins appeared lower in 40 Hz than random groups (*t*_(8)_ = 1.415, *p* = 0.1948, unpaired *t* test; Fig. 3*D*,*G*). These results show that 40 Hz flicker transiently upregulates the NF-κB pathway after ∼5–15 min and that this difference in upregulation is no longer detectable after 60 min of flicker.

We found that MAPK phosphorylation patterns were similar to those of NF-κB but with different kinetics. MAPK phospho-signaling was significantly different between 40 Hz and random groups after 60 min of flicker but not after 5 or 15 min. Phosphorylation of MAPK proteins following 5 min of 40 Hz flicker did not significantly differ from random stimulation, as indicated by no significant LV1 separation (*t*_(32)_ = 1.870, *p* = 0.0707, unpaired *t* test; Fig. 4*B*,*E*). While phosphorylation of MAPK proteins also did not significantly differ following 15 min of 40 Hz or random flicker (*t*_(8.582)_ = 1.99, *p* = 0.2624, Welch's corrected unpaired *t* test), the heatmap of phosphoprotein levels showed a trend for more phosphorylated MAPK proteins in the 40 Hz group than in the random group (Fig. 4*C*,*F*). MAPK phospho-signaling did significantly differ between 40 Hz and random stimulation following 60 min of flicker exposure, as revealed by significantly separated LV1 scores (*t*_(10)_ = 3.709, *p* = 0.004, unpaired *t* test; Fig. 4*D*,*G*). This effect was mostly driven by differences in phosphorylated MSK1, and MEK1 (Fig. 4*J*). These results show that 40 Hz flicker upregulates the MAPK pathway after ∼60 min.

### Correlations between phosphorylation of proteins increases after minutes of 40 Hz flicker

**Figure 5.**
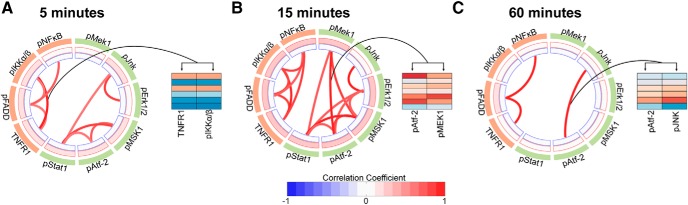
Phosphoprotein network correlations are highest after 15 min of 40 Hz flicker exposure. ***A***, Significant correlations following 5 min of 40 Hz flicker in NF-κB (orange) and MAPK (green) phosphoprotein levels across animals displayed as links, with color indicating the strength of positive correlations (red) or negative correlations (blue). No negative correlations were found in these interactions. *q* < 0.1, false discovery rate correction for 135 comparisons, Pearson correlation test. Right, Example of correlated phosphoproteins. ***B***, As in ***A*** for 15 min of 40 Hz flicker. ***C***, As in ***A*** for 60 min of 40 Hz flicker.

Because phospho-signaling is transient, another way of assessing activity in these pathways is to measure coordinated phosphorylation among proteins. We thus hypothesized that the activation of these pathways would lead to coordinated phosphoprotein levels at time points before our observed increases in cytokine expression. Therefore, we examined how coordination both within and between NF-κB and MAPK networks changed over the course of 40 Hz flicker exposure. To do so, we used variability across animals and investigated protein covariation across animals by calculating the correlation coefficients of each protein pair from NF-κB and MAPK pathways after 5, 15, and 60 min of 40 Hz flicker stimulation (Eisen et al., 1998; Kanonidis et al., 2016; Fig. 5). In other words, this analysis revealed protein phosphorylation levels that significantly increased or decreased together across animals (Fig. 5; *q* < 0.1, false discovery rate correction for 135 comparisons, Pearson correlation test).

Over the time points assessed, we found the number of significant correlations between proteins within each pathway was highest after 15 min of 40 Hz flicker. After 5 min of 40 Hz flicker, five proteins were significantly correlated within the NF-κB pathway and four within the MAPK pathway (Fig. 5*A*). After 15 min of 40 Hz flicker, more protein correlations were found: 6 significant correlations in the NF-κB pathway, and 10 significant correlations in the MAPK pathway (Fig. 5*B*). Interestingly, the proteins that were significantly correlated after 15 min but not 5 min of flicker were farther downstream in the MAPK pathway: pERK1/2, pMSK1, pATF-2, and pSTAT1 (Fig. 4*A*). After 60 min of 40 Hz flicker, only three significant correlations were found in both pathways (Fig. 5*C*). Of note, all significant correlations were within and not across NF-κB and MAPK pathways in all time points assessed.

### Animal behavior is similar across flicker conditions

**Figure 6.**
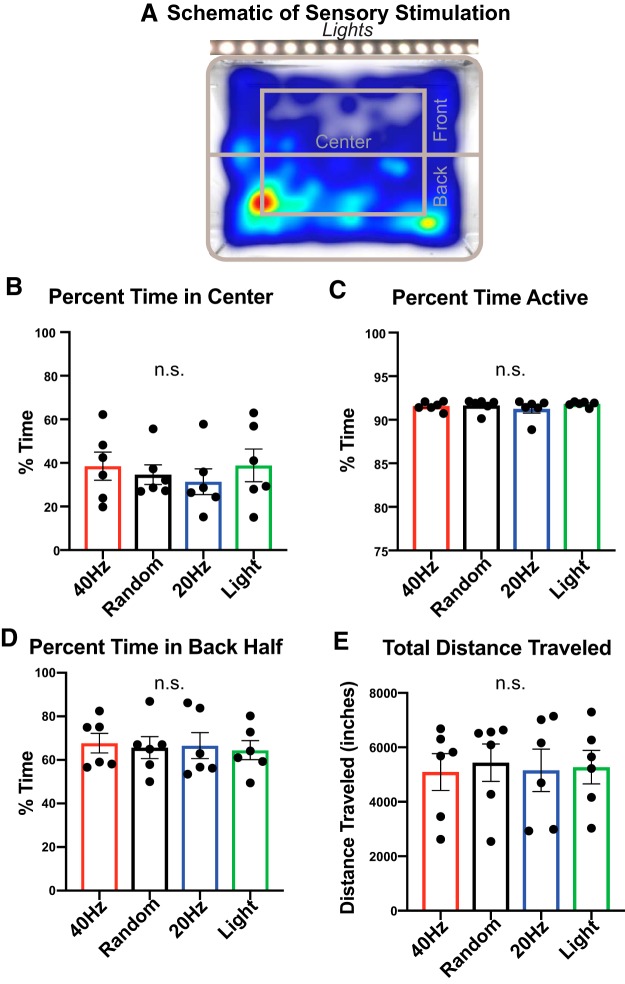
Animal behavior is similar across conditions during exposure. ***A***, Example heatmap representing the amount of time spent in locations within the flicker enclosure. Delineations indicating the center, front, and back of the cage that were used for behavior analysis. ***B***, Total percentage of time animals in each group spent in the center of the cage (*F*_(3,15)_ = 0.8754, *p* = 0.4757, RM-ANOVA). Error bars indicate the mean ± SEM. Five animals total were used, represented by dots indicating individual animals for all bar graphs in this figure (red = 40 Hz, black = random, blue = 20 Hz, green = light). ***C***, Total percentage of time animals in each group spent active (*F*_(3,15)_ = 0.9306, *p* = 0.4502, RM-ANOVA). ***D***, Total percentage of time animals in each group spend in the back half of the cage (*F*_(3,15)_ = 0.1727, *p* = 0.9132, RM-ANOVA). ***E***, Total distance animals in each group traveled during stimulation (*F*_(3,15)_ = 0.3392, *p* = 0.7973, RM-ANOVA).

Given our findings indicating cytokine and phosphoprotein upregulation induced by 40 Hz flicker, we sought to determine whether behavioral differences during flicker could explain these results. We assessed both overall activity levels, measured as the percentage of time active and the total distance traveled, as well as exploratory versus anxiety-like behavior, assessed by the percentage of time animals spent in the center of the environment versus along the walls (perimeter). More anxious animals stay close to the walls of an enclosure. We recorded mice during 1 h of either 40 Hz flicker, random flicker, 20 Hz flicker, or light and tracked mouse movement (Fig. 6*A*). Our results revealed no significant differences between different flicker conditions in the amount of time spent in the center versus the perimeter of the enclosure, time spent active instead of freezing, time spent in the front (near the light source) versus the back half of the enclosure, and total distance traveled [center vs perimeter: (Fig. 6*B*) *F*_(3,15)_ = 0.8754, *p* = 0.4757, RM-ANOVA; activity: (Fig. 6*C*) *F*_(3,15)_ = 0.9304, *p* = 0.4502, RM-ANOVA; front vs back: (Fig. 6*D*) *F*_(3,15)_ = 0.1727, *p* = 0.9132, RM-ANOVA; distance: (Fig. 6*E*) *F*_(3,14)_ = 0.3392, *p* = 0.7973, RM-ANOVA). These results show that different visual stimulation conditions, including different frequencies of flicker, periodic or aperiodic flicker, or constant or flickering light stimuli, do not differentially affect animal behavior or induce an anxiety-like phenotype. Thus, the molecular changes we found in response to different visual stimulation conditions are not due to changes in animal behavior.

### 40 Hz flicker induces neuroimmune profile distinct from acute inflammation

**Figure 7.**
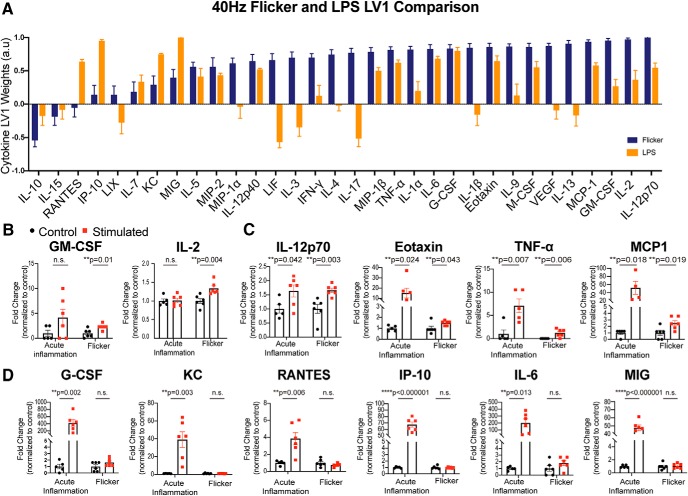
The 40 Hz flicker cytokine response differs from LPS-induced acute inflammation. ***A***, The weighted profile of cytokines that make up LV1 following 40 Hz flicker (blue) or LPS (orange; mean ± SD from a leave-one-out cross-validation, *N* = 6). ***B***, GM-CSF and IL-2 expression levels significantly differed in 40 Hz versus random (“Flicker”) but not LPS versus vehicle (“Acute Inflammation”). Error bars indicate the mean ± SEM. The *p* values for *t* test comparisons between groups are listed in the figure. ***C***, IL-12p70, Eotaxin, TNF-α, and MCP1 expression levels significantly differed for both 40 Hz versus random and LPS versus vehicle. Error bars indicate the mean ± SEM. The *p* values for *t* test comparisons between groups are listed in figure. ***D***, G-CSF, KC, RANTES, IP-10, IL-4, and MIC expression levels significantly differed for LPS versus vehicle but not for 40 Hz versus random flicker. Error bars indicate the mean ± SEM. The *p* values for *t* test comparisons between groups are listed in figure.

Next, we wondered how the cytokine profile in response to 40 Hz flicker differed from acute inflammation. Thus, we assessed cytokine expression in the visual cortex of animals intraperitoneally injected with LPS, a traditional, acute model of inflammation (Gabellec et al., 1995; Qin et al., 2007; Meneses et al., 2018). As in our flicker experiments, we characterized cytokine expression in visual cortex of mice injected with LPS versus vehicle injection and separated these differences using a PLSDA (Fig. 7). The latent variable that best separated LPS- and vehicle-injected animals (Fig. 7, LV1, orange) was most heavily weighted by expression of MIG, IP-10, IL-3, and IL-17 (Fig. 7*A*). The profile of cytokine weights that separated LPS from vehicle-injected animals (Fig. 7*A*, LV1, orange) differed from the profile that separated 40 Hz-exposed versus random-exposed animals (Fig. 7*A*, LV1, blue). These results show that 40 Hz flicker and LPS stimulation lead to distinct immune profiles.

We further analyzed the differences between LPS and 40 Hz flicker by comparing individual cytokines following LPS or 40 Hz flicker relative to their controls. These comparisons revealed that most cytokines differed between LPS and 40 Hz flicker, both in terms of whether or not each cytokine was modulated by the stimulus and in terms of the magnitude of the response relative to control (Fig. 7*B–D*). For example, GM-CSF and IL-2 significantly differed between animals exposed to 40 Hz flicker versus random stimulation but did not differ between animals injected with LPS or vehicle (40 Hz vs random: GM-CSF, *t*_(10)_ = 3.114, *p* = 0.011; IL-2, *t*_(10)_ = 3.613, *p* = 0.0047; LPS vs vehicle: GM-CSF, *t*_(9)_ = 1.646, *p* = 0.134; IL-2, *t*_(9)_ = 0.167, *p* = 0.871; Fig. 7*B*). In contrast, G-CSF, KC, MIG, RANTES, IP-10, and IL-6 significantly differed between LPS and vehicle but not between 40 Hz and random (LPS vs vehicle: G-CSF, *t*_(9)_ = 4.069, *p* = 0.003; KC, *t*_(9)_ = 3.883, *p* = 0.004; MIG, *t*_(9)_ = 12.16, *p* < 0.000001; RANTES, *t*_(9)_ = 3.502, *p* = 0.007; IP-10, *t*_(9)_ = 14.86, *p* < 0.000001; IL-6, *t*_(9)_ = 3.107, *p* = 0.013; 40 Hz vs random: G-CSF, *t*_(10)_ = 1.661, *p* = 0.128; KC, *t*_(10)_ = 0.450, *p* = 0.662, MIG, *t*_(10)_ = 0.2928, *p* = 0.776; RANTES, *t*_(10)_ = 1.335, *p* = 0.212; IP-10, *t*_(10)_ = 0.3421, *p* = 0.739; IL-6, *t*_(10)_ = 1.437, *p* = 0.1813; Fig. 7*C*). Last, there was a subset of cytokines, IL-12p70, Eotaxin, TNF-α, and MCP1, that significantly differed in both LPS versus vehicle and 40 Hz versus random (LPS vs vehicle: IL-12p70, *t*_(9)_ = 2.359, *p* = 0.043; eotaxin, *t*_(9)_ = 2.710, *p* = 0.024; TNF-α, *t*_(9)_ = 3.561, *p* = 0.007; MCP1, *t*_(9)_ = 2.884, *p* = 0.018; 40 Hz vs random: IL-12p70, *t*_(10)_ = 3.729, *p* = 0.004; eotaxin, *t*_(10)_ = 2.318, *p* = 0.043;TNF-α, *t*_(10)_ = 3.512, *p* = 0.006; MCP1, *t*_(10)_ = 2.785, *p* = 0.019; Fig. 7*D*). For those cytokines that significantly increased expression in response to both LPS and 40 Hz stimulations relative to controls, the scale of the difference between the two comparisons was usually much larger when comparing LPS versus vehicle. For example, the difference in expression of MCP1 was >10-fold larger between LPS and vehicle than between 40 Hz and random, though both differences were significant (Fig. 7*D*). Overall, these results show that acute inflammation via LPS and 40 Hz flicker produce different immune responses.

## Discussion

In this study, we used multiplexed immunoassays to profile the expression of cytokines and phospho-signaling within key pathways that regulate the immune response of the brain to different forms of visual stimulation. We found that 40 Hz light flicker rapidly and transiently stimulated phospho-signaling within the NF-κB pathway in mouse visual cortex. NF-κB phospho-signaling was followed by increased phospho-signaling within the MAPK pathway and increased the expression of a diverse profile of cytokines involved in microglial recruitment (e.g., M-CSF), neurotrophic properties (e.g., IL-6), and synaptic plasticity (e.g., IL-1). Furthermore, we found distinct cytokine responses to different visual stimuli, including 20 Hz flicker, random flicker, and constant light. These results show that specific frequencies and patterns of visual stimulation lead to different cytokine responses. Because these assays were performed in wild-type animals, the results reveal that 40 Hz flicker stimulation modulates immune function independent of disease pathology. Thus, these findings suggest that this approach could be applied to many diseases that affect brain immune function. Importantly, we found that 40 Hz flicker induced a cytokine signature that differs from an LPS model of inflammation. Together, our data show that visual flicker rapidly activates canonical intracellular signaling pathways and induces a unique cytokine expression profile. Due to the multifunctional nature of these pathways and the broad range of cytokines expressed, the effects of visual flicker likely extend beyond neural immune activity to changes in neuronal health, synaptic plasticity, and other functions.

### 40 Hz flicker induces a unique cytokine profile in the visual cortex

Because nothing is known about the effect of gamma oscillatory activity on cytokine expression levels, we used a multiplex approach to determine the global impact of this activity on the main immune signals of the brain. Our results establish that 40 Hz flicker upregulated a combination of cytokines in the visual cortex resulting in a neuroimmune profile distinct both from other types of flicker stimulation and from acute inflammation induced by LPS. Interestingly, LV1 better separated 40 Hz stimulation from 20 Hz and light than from random stimulation. This point is reinforced by our finding that the top LV1-contributing cytokines did not differ significantly between 40 Hz and random stimulation. However, other cytokines, such as IFN-γ, IL-12p70, and GM-CSF, showed significant differences between 40 Hz and random stimulation (Fig. 1*G*). Thus, random stimulation leads to an immune profile with an overlap in the expression of some cytokines to that of 40 Hz but distinct expression levels of other cytokines (Fig. 2). While random stimulation does not induce gamma oscillations, randomized pulses of light slightly alter neural activity in visual cortex over a much broader range of frequencies than gamma frequency light flicker (Iaccarino et al., 2016; Singer et al., 2018). Therefore, random flicker has some similar and some different effects on cytokines than 40 Hz flicker, but across multiple cytokines 40 Hz flicker produces a distinct immune profile.

Random flicker, 20 Hz flicker, and light stimulation each induce the expression of unique subsets of cytokines (Fig. 2). When comparing cytokine expression, LV1 best separated 40 Hz stimulation from the other controls. In contrast, LV2 best separated all of the control groups from each other. Interestingly, while LV1 separated animals stimulated with 20 Hz and light stimulation from those stimulated with 40 Hz visual flicker, LV2 did not separate these groups as well. In fact, LV2 best separated animals stimulated with random visual flicker from other groups. When examining individual cytokines, plotted in Figure 2, there is a significant difference in the expression of IL-10 and RANTES between 40 and 20 Hz flicker, and a significant difference in the expression of IL-10, IL-15, and RANTES between 40 Hz flicker and light. Together, these results demonstrate that 40 Hz stimulation leads to increased expression of the many cytokines in the visual cortex compared with other conditions, but each of our control stimulations produces its own unique profile of cytokine expression.

### 40 Hz flicker-induced cytokines have neuroprotective functions

We found that 40 Hz flicker is upregulating a unique combination of cytokines, and we hypothesize that no one cytokine in particular is responsible for neuroprotective function, but rather the combinatory profile is necessary. This effect would coincide with what is known about gamma oscillations, that no one neuron is responsible for gamma oscillations, but rather the combination of neuronal activity.

By focusing on the functions of the top five cytokines distinguishing 40 Hz flicker from all other groups M-CSF, IL-6, MIG, IL-4, and KC (also known as CXCL1), it becomes apparent that the unique immune function of each cytokine may contribute to an overall neuroprotective effect (Fig. 1). M-CSF (colony stimulating factor 1) transforms microglia morphologically and has been reported to enhance phagocytosis of amyloid-β (Imai and Kohsaka, 2002; Mitrasinovic and Murphy, 2003). Furthermore, the primary receptor for M-CSF, CSF-1R, is primarily expressed by microglia in the brain (Elmore et al., 2014). Thus, M-CSF may be responsible for previously observed morphological transformation of microglia in 5XFAD mice (after 1 h of 40 Hz light flicker stimulation followed by 1 h of no stimulation) because those microglial changes are associated with engulfment (Iaccarino et al., 2016).

Interestingly, IL-6, the second top cytokine that increased following 1 h of 40 Hz flicker, is a pleiotropic cytokine with both protective and pathogenic effects. The potential pathogenic effects of IL-6 include stimulating amyloid precursor protein expression (Ringheim et al., 1998). In contrast, the neuroprotective effects of IL-6 include increasing synaptic density and inhibiting macrophage expression of TNF-α, a cytokine known to have proinflammatory and neurotoxic properties (Decourt et al., 2017). In our own work, increased expression of IL-6 was correlated with a resiliency to Alzheimer's pathology, defined as human subjects with no neuronal loss or cognitive impairment even in the presence of plaques and tangles associated with Alzheimer's (Barroeta-Espar et al., 2019). IL-6 is also important for neural progenitor cell health as IL-6 knockout significantly reduced neural progenitor cell density in multiple brain regions (Bowen et al., 2011). Furthermore, IL-6 levels correlate with improved learning and memory performance in both animals and humans (Baier et al., 2009; Donegan et al., 2014; Wang et al., 2017).

The third flicker-upregulated cytokine, MIG, is a chemokine involved in microglial recruitment via its receptor, CXCR3 (Rappert et al., 2004). In brain endothelial cells *in vitro*, MIG is released in response to the presence of other cytokines (Ghersa et al., 2002). It is specifically responsible for recruiting T cells to the brain and responds to IFN-γ, suggesting that MIG may be released to combat infection. Indeed, injecting mice with an anti-MIG treatment leads to increased viral infection and pathology (Ure et al., 2005).

Finally, the fourth and fifth top cytokines, IL-4 and KC (CXCL1) signal to microglia (Johnson et al., 2011; De Filippo et al., 2013; Fenn et al., 2014). IL-4 has been shown to reprogram microglia to stimulate neurite outgrowth after spinal cord injury (Fenn et al., 2014). In addition, adeno-associated virus-mediated overexpression of IL-4 in APP mice reduced Aβ plaque load (Latta et al., 2015). KC (CXCL1) is a chemokine involved in recruiting neutrophils, a type of white blood cell, signaling to microglia (Johnson et al., 2011; De Filippo et al., 2013). Finally, KC signals to the receptor CXCR2, which is expressed on microglia and has been reported to mediate microglial recruitment (Lee et al., 2012). In total, our data show that 40 Hz light flicker stimulates the expression of diverse cytokines with both immunomodulatory and neuroprotective properties.

It is important to note that our results were based on experiments performed with male mice. We chose to focus on males because we wanted to assess how 40 Hz flicker, which induces gamma oscillations, impacts neuroimmune signaling, building on previous articles that have studied this effect only in male mice (Iaccarino et al., 2016; Adaikkan et al., 2019; Martorell et al., 2019). However, we acknowledge that this is a limitation of this study because there are effects of sex on immune response, and ovarian hormones have been shown to impact cytokine expression (Arakawa et al., 2014; Cai et al., 2016). Future studies should further explore how this effect may differ between the sexes.

### 40 Hz flicker cytokine response differs from acute inflammation

We found that 40 Hz flicker upregulates a profile of cytokines in a short period of time, similar to an acute proinflammatory stimulus. However, previous work has shown that 40 Hz flicker has a neuroprotective effect. Therefore, we wanted to compare how 40 Hz flicker induced cytokine expression differs from a known proinflammatory stimulus operating under a similar acute time period.

Our analysis showed that 40 Hz flicker stimulates the expression of diverse cytokines that differs from a model of acute inflammation. This difference was established by comparing the LV1 cytokine profile of gamma sensory stimulation and acute LPS inflammation. While there may be an overlap in some cytokines that were elevated, the overall profiles are distinct between these two stimulations due to the differences in magnitude of responses and the overall cytokines included. Two cytokines, GM-CSF and IL-2, significantly differed between 40 Hz flicker and random stimulation but did not differ between LPS and vehicle administration. The specific response of these two cytokines to 40 Hz flicker is especially interesting because GM-CSF may be both neuroprotective and proinflammatory and low-dose IL-2 rescues cognitive and synaptic plasticity deficits in a mouse model of AD (Bhattacharya et al., 2015; Alves et al., 2017; Kiyota et al., 2018). Furthermore, the combination of GM-CSF and IL-2 has been suggested to have synergistic effects. The combination of these cytokines has been used to prevent immune dysregulation in multiple models of disease (Baiocchi et al., 2001; Stagg et al., 2004; Elias et al., 2005). Thus, the combination of these two cytokines following 40 Hz flicker may be neuroprotective.

Some cytokines significantly differed between both 40 Hz versus random flicker and LPS versus vehicle administration. An interesting observation from our data is that while there was overlap in some of these cytokines, there was a noticeable difference in the scale of separation between LPS and vehicle versus 40 Hz and random stimulation. While both were significantly upregulated by stimulation, LPS had a much larger effect when compared with the control condition. The magnitude of the cytokine response has been suggested to alter the effects of a cytokine (Saadoun et al., 2011; Shachar and Karin, 2013). Thus, this low-dose increase caused by 40 Hz flicker may be more responsible for the neuroprotective effect of 40 Hz flicker.

Last, IL-12p70, the bioactive form of the p35 and p40 combined IL-12 subunits, was upregulated in both LPS and 40 Hz versus controls. In contrast, IP-10, which is downstream of IL-12, was significantly different between LPS and control but not 40 Hz and random. IP-10 is induced by IFN-γ, which is a proinflammatory cytokine downstream of IL-12 (Sgadari et al., 1996). This finding suggests that while both LPS and 40 Hz flicker may upregulate some similar cytokines, they may be leading to different downstream mechanisms that explain why LPS is known to be neurotoxic, while 40 Hz flicker is known to be neuroprotective (Iaccarino et al., 2016; Martorell et al., 2019).

### 40 Hz flicker induces neuroimmune intracellular signal transduction pathways

Our data show that 40 Hz flicker stimulates NF-κB and MAPK pathway signaling, which increased following 15 or 60 min of 40 Hz flicker, respectively, followed by the expression of cytokines. These findings are consistent with the known transient kinetics of phospho-signaling and downstream gene and protein expression (Janes et al., 2005; Gierut et al., 2015). While the effect of 5 min of 40 Hz flicker on the NF-κB pathway were not significant, many of the animals did separate on LV1 and this was mostly driven by increases in TNFR1 and phosphorylated FADD. Interestingly, these two proteins are upstream in the NF-κB pathway, and therefore we would expect these to become activated earlier than other proteins in the pathway. After 15 min of 40 Hz flicker, all proteins in the NF-κB pathway were up-regulated. Thus, we conclude that signaling through the NF-κB pathway may begin after ∼5 min of 40 Hz flicker in some animals, but the full effects may not be detectable until after 15 min of 40 Hz flicker. A similar trend is observed for the MAPK pathway, but it seems to be later, where 15 min of 40 Hz flicker begins to upregulate the pathway and 60 min shows a distinct separation between 40 Hz flicker and random groups. Later onset of MAPK pathway activation may be related to increased expression of cytokines that act through the MAPK pathway.

For phosphoprotein analysis, we specifically compared 40 Hz flicker and random flicker exposure. We chose random flicker as our control because this stimulation type controls for the largest number of stimulation variables. However, we anticipate that if we compared 40 to 20 Hz stimulation instead, we would see a bigger difference between groups, since 20 Hz had a more reduced cytokine expression profile in Figure 1. In contrast, we anticipate that light stimulation may also upregulate signaling pathways. We expect that because light stimulation uniquely upregulates some cytokines, distinct from 40 Hz flicker, that light may also upregulate some of the signaling pathways that are known to regulate cytokines.

Interestingly, our data show that the NF-κB pathway is downregulated below random flicker after 1 h of 40 Hz flicker stimulation, suggesting that the effects of 40 Hz flicker on these pathways are transient. Both NF-κB and MAPK pathways are highly regulated via feedback mechanisms (Hutti et al., 2007; Lake et al., 2016). Given that our observed changes are occurring over the course of an hour, negative feedback mechanisms within the pathways are likely responsible for the downregulation of these pathways.

Our hypothesized time course of pathway activity is further supported when examining correlations between proteins. After 5 min of 40 Hz flicker, a pattern emerged within each pathway. Specifically, in the NF-κB pathway, both pFADD and TNFR1 levels were highly correlated aligning with increased quantities of these two proteins at the same time course. TNFR1 is upstream in the NF-κB signaling pathway, suggesting that 5 min of flicker begins to modulate the top of the pathway. Fifteen minutes of 40 Hz flicker revealed more correlations among proteins, and these correlations included more proteins downstream in the pathway, suggesting that the pathway is progressively activated with longer flicker exposure. However, after 60 min of 40 Hz flicker, many correlations were no longer present. In fact, in the MAPK pathway, only pATF-2 and pJnk were correlated, downstream in the pathway. These correlation results help support our activation data. However, we note that the small sample size (six animals), might account for the low significance associated with 60 min of stimulation at 40 Hz. Further, while the analysis was designed to reveal correlations between any proteins, all significant correlations remained within each pathway, supporting the idea that each pathway is operating with independent dynamics.

Both the NF-κB and MAPK pathways play key roles in immune function, synaptic plasticity, and learning and memory. NF-κB and MAPK pathways regulate cytokine levels by activating transcription factors involved in cytokine expression (Hommes et al., 2003; Cloutier et al., 2007; Liu et al., 2017). Increased phosphorylation in the MAPK pathway occurs after spatial memory and is necessary for long-term memory formation (Blum et al., 1999). The NF-κB pathway is more traditionally known for its role in inflammation, but is also necessary for long-term potentiation and long-term depression and is increased following synaptic stimulation (Albensi and Mattson, 2000; Meffert et al., 2003).

The 40 Hz light flicker likely stimulates intracellular phospho-signaling and the expression of diverse genes by driving neural activity. Several studies have shown that flickering light at a particular frequency drives that frequency neural activity in primary visual areas and 40 Hz flicker induces neural activity ∼40 Hz (Gray et al., 1989; Iaccarino et al., 2016; Singer et al., 2018). Neuronal activity can lead to calcium influx, which, in turn, stimulates multiple molecular signaling events, including the activation of multiple isoforms of protein kinase C, which can activate the NF-κB and MAPK pathways. In fact, the inhibition of calcium activity blocks NF-κB activation (Meffert et al., 2003). These pathways provide a possible link among gamma frequency activity induced by 40 Hz flicker, phospho-signaling, and downstream gene expression.

### Behavior is similar across different visual stimulation conditions

Cytokine expression can be modified by the behavioral state of an animal, for example when an animal is under stress (Minami et al., 1991; Ishikawa et al., 2001). While it is known that chronic 40 Hz flicker does not affect anxiolytic behaviors over time, we wanted to ensure that 40 Hz flicker stimulation did not differentially affect animal behavior in real time over the timescales of our analyses when compared with other stimulation types (Martorell et al., 2019). We measured the total time spent in the center versus the surround areas of the enclosure and the time spent active versus freezing. We concluded that flicker stimulation did not invoke anxiety-like behavior or changes in overall activity levels. To assess whether one stimulation type was more aversive than others, we analyzed the time spent in the front of the cage where the lights were positioned versus the back and found no differences across stimulation conditions. Interestingly, animals across all conditions spent somewhat more time (∼65%) in the back of the cage, which is not surprising considering that mice tend to stay in dark areas if given the choice (Crawley and Goodwin, 1980). Last, we measured the distance animals traveled within each stimulation type and found no differences across flicker conditions. In short, our analysis revealed no differences in any of our measured animal behaviors between stimulation conditions. Therefore, our observations show that induced phospho-signaling and cytokine expression after 40 Hz light flicker cannot be explained by changes in animal behavior.

In total, our results show that 40 Hz flicker drives rapid signaling within the NF-κB and is followed by activation of the MAPK pathway and an upregulation of cytokines. The diverse functions regulated by these pathways together with the diversity of cytokine and growth factors expressed in response to 40 Hz stimulation suggests that minutes of flicker stimulation may induce changes in various cell and tissue functions that include immune activity and neuronal and synaptic health. Furthermore, different forms of visual stimulation induced unique cytokine profiles. Thus, flicker stimulation may be used to rapidly and noninvasively manipulate the signaling and expression of genes that go beyond neural immune activity. Importantly, all our signaling analyses were conducted in wild-type animals, helping establish the effects of 40 Hz flicker stimulation independent of disease pathology. Our work provides a foundation for testing the therapeutic potential of the use of flicker in other disorders involving the neuroimmune system.
